# EGFR-mutated lung cancer: a paradigm of molecular oncology

**DOI:** 10.18632/oncotarget.186

**Published:** 2010-10-25

**Authors:** Zhenfeng Zhang, Amy L. Stiegler, Titus J. Boggon, Susumu Kobayashi, Balazs Halmos

**Affiliations:** ^1^ Division of Hematology/Oncology, Herbert Irving Comprehensive Cancer Center, New York Presbyterian Hospital- Columbia University Medical Center, New York, NY, USA; ^2^ Department of Pharmacology, Yale School of Medicine, New Haven, CT, USA; ^3^ Division of Hematology/Oncology, Beth Israel Deaconess Medical Center, Harvard Medical School, Boston, MA, USA

**Keywords:** EGFR, tyrosine kinase, lung cancer, therapy, oncology

## Abstract

The development of EGFR tyrosine kinase inhibitors for clinical use in non-small cell lung cancer and the subsequent discovery of activating EGFR mutations have led to an explosion of knowledge in the fields of EGFR biology, targeted therapeutics and lung cancer research. EGFR-mutated adenocarcinoma of the lung has clearly emerged as a unique clinical entity necessitating the routine introduction of molecular diagnostics into our current diagnostic algorithms and leading to the evidence-based preferential usage of EGFR-targeted agents for patients with EGFR-mutant lung cancers. This review will summarize our current understanding of the functional role of activating mutations, key downstream signaling pathways and regulatory mechanisms, pivotal primary and acquired resistance mechanisms, structure-function relationships and ultimately the incorporation of molecular diagnostics and small molecule EGFR tyrosine kinase inhibitors into our current treatment paradigms.

## HER FAMILY/EGFR- BACKGROUND/ROLE IN CANCER

The epidermal growth factor receptor (EGFR) family, a member of the subclass I of the transmembrane receptor tyrosine kinase superfamily, consists of four closely related members: EGFR/ERBB1/HER1, ERBB2/HER2, ERBB3/HER3, and ERBB4/HER4 [1]. The founder member, EGFR was first identified as a 170-kDa protein on the membrane of A431 epidermoid cells and other ERBB members were identified by screening of cDNA libraries for EGFR related molecules [2,3]. These receptors are normally expressed in various tissues of epithelial, mesenchymal, and neural origin. The crucial roles of the EGFR family proteins are supported by a series of knockout mouse studies. Mice lacking EGFR die between day 11.5 of gestation and day 20 after birth, depending on their genetic backgrounds [4]. Analyses of the knockout mice reveal placental defects and lung immaturity, both of which can be the causes of death. They also show abnormalities in the bone, brain, heart, and various epithelial organs such as gastrointestinal tract, skin, hair follicles and eyes [4]. Detailed analyses show that deletion of EGFR leads to impaired branching and deficient alveolization and septation in lungs [5]. In addition, type II pneumocytes are immature, and there is a lack of response in up-regulation of surfactant protein C in mice lacking EGFR [5]. Mice lacking ERBB2 , ERBB3, or ERBB4 are embryonic lethal and have defects in cardiac and neuronal development [4]. In mammals, eleven growth factors bind to the ERBB receptors: EGF, transforming growth factor α (TGFα), heparin-binding EGF-like growth factor, amphiregulin, beta-cellulin, epiregulin, epigen, and neuregulin1-4, of which seven are ligands of EGFR [6,7]. Upon binding, the ERBB receptors form homo- or hetero-dimers, resulting in autophosphorylation of the receptors. Of note, mice lacking EGF show no overt phenotype [8]. Mice lacking TGFα show hair follicle, skin, and eye abnormalities, however, they are viable and fertile [9,10]. These observations indicate that there is a high level of redundancy among ligands.

Given the pivotal roles of the ERBB receptors in normal development, one can imagine that dysregulation of these genes or proteins can lead to tumorigenesis. Indeed, EGFR is overexpressed in a variety of human cancers including lung, head and neck, colon, pancreas, breast, ovary, bladder and kidney, and gliomas [11,12]. More than 60% of non-small cell lung cancers (NSCLCs) show EGFR overexpression, whereas no overexpression is detected in small cell lung cancer [13]. The overexpression of EGFR is presumably caused by multiple epigenetic mechanisms, gene amplification, and oncogenic viruses [11]. It has been shown that EGFR expression is associated with poor prognosis [14]. In addition to EGFRs themselves, the EGFR ligands may also play an important role in lung tumorigenesis. EGF, TGFα, and amphiregulin are expressed in NSCLCs, and activate EGFR and its downstream signaling pathways by autocrine loops [15]. In addition, a distinct ligand for ERBB3 and ERBB4, called neuregulin-1 is overexpressed in NSCLC [15].

## EGFR MUTATIONS DISCOVERY/BIOCHEMISTRY

**Figure 1:**
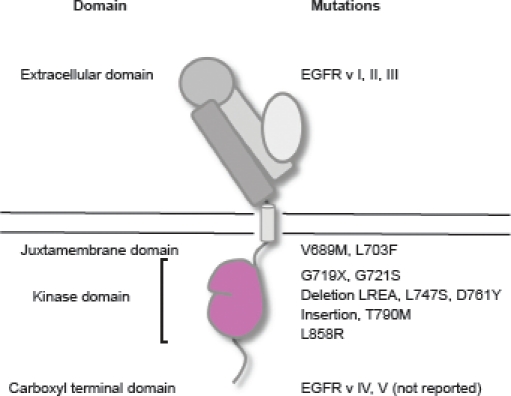
Oncogenic EGFR variants Cartoon shows the positions of key EGFR mutations/variants in the corresponding domains.

The EGFR protien consists of three regions: an extracellular ligand-binding region, a single transmembrane helix region, and a cytoplasmic region. The tyrosine kinase domain accounts for approximately 50% of the cytoplasmic region, with the remainder composed of the 38 amino acid cytoplasmic juxtamembrane (JM) region and the 225 amino acid carboxyl terminal (CT) region [16]. As shown in Figure 1, mutations in the EGFR gene cluster in specific areas, suggesting that these areas are crucial for receptor function or regulation.

### Mutations in the extracellular region

It has been shown that there are three major types of deletion mutations in the extracellular region depending on the site and length of deletions: EGFR vI, EGFR vII, and EGFR vIII. They were originally discovered in gliomas [17]. Of these mutant forms, EGFR vIII is the most common mutation in gliomas (30-50%) and has been extensively studied since its discovery in 1990 [3]. This mutant lacks a large part of the extracellular portion including the ligand-binding region, leading to constitutive dimerization and activation of the receptor. This mutation is detected in 5% of lung squamous cell carcinomas, but not in other non-small cell histologies [18]. In addition to the deletions, novel missense mutations in the extracellular domain have been reported in 13.6% in glioblastomas; however, these point mutations have not been found in lung tumors with any frequency [19].

### Mutations in the juxtamembrane region

A recent study revealed that there is a domain in the EGFR juxtamembrane region that plays an activating role. This JM activating domain seems to enhance formation of the asymmetric dimer, thereby promoting allosteric activation of the acceptor kinase domain (see “Structural Implicatons” section below). Several rare mutations in this domain have been identified in NSCLC patients. Two of these missense mutants, V689M and L703F are constitutively active, possibly because they stabilize acceptor/donor interactions [16].

### Mutations in the kinase domain

A tandem kinase duplication in the tyrosine kinase domain has been described in glioblastomas. This mutant is constitutively active and confers tumorigenicity [20]. In lung cancer, a series of mutations in the kinase domain was originally identified in correlation with sensitivity to EGFR inhibitors. Two anilinoquinazoline EGFR tyrosine kinase inhibitors (TKIs), gefitinib and erlotinib, were approved for use in unselected patients with NSCLC in the 2^nd^ and 3^rd^ line setting after failure of first line platinum-based chemotherapy in 2003 and 2004, respectively in the United States [21] and some patients were noted to have major and sometimes durable responses [22]. The selective response of a fraction of NSCLC to these agents can be explained by somatic mutations in the tyrosine kinase domain of EGFR in most patients with NSCLC responsive to gefitinib or erlotinib [23-25]. EGFR mutations are more common in NSCLC from tumors with adenocarcinoma histology, women, Asians, and never smokers with widely varying frequencies dependent on the population examined [26-28]. EGFR mutations are rarely found in squamous cell carcinomas of the lung, small cell lung cancer or other epithelial malignancies. Thus, activating somatic EGFR mutations are a unique feature of a sub-class of NSCLC. The most prevalent EGFR mutations consist of small inframe deletions around the conserved LREA motif of exon 19 (residues 747-750) and a point mutation (L858R) in exon 21 [21], which account for more than 90% of all EGFR kinase mutations. Oncogenecity of the exon 19 deletion and the L858R mutation has been shown in inducible mouse models [29,30]. Point mutations in exon 18, predominantly at G719 account for approximately another 5% of EGFR mutations [15]. In-frame insertions and point mutations in exon 20 account for 5% of the mutations, which are rather insensitive to reversible EGFR inhibitors but might be sensitive to irreversible EGFR inhibitors, such as CL-387,785 [15,31]. These EGFR mutations activate the EGFR signaling pathway and promote EGFR-mediated pro-survival and anti-apoptotic signals through down-stream targets as discussed below. In contrast to the activating mutations above, a secondary mutation was identified as a single base pair change leading to a threonine to methionine (T790M) amino acid alteration in exon 20 as a mechanism of acquired resistance to EGFR inhibitors. It accounts for more than 50% of primarily EGFR TKI-sensitive lung tumors which become resistant to EGFR inhibitors[32,33]. Other resistance mutations in exon 19, such as D761Y and L747S, have been reported [34,35]; however, these mutations seem to be rare. These EGFR kinase domain mutations and other kinase mutations such as K-RAS mutations usually exhibit a mutually exclusive pattern in NSCLC, suggesting that the EGFR kinase mutations per se are responsible for initiating tumors.

In gliomas, two forms of deletion mutants in the carboxyl terminal region have been reported. EGFR vIV harbors an in-frame deletion and EGFR vV has a carboxyl terminal truncation [17](Fig. 1). These mutants seem to be constitutively active: computational analyses suggest that this is due to the fact that the deleted region has an inhibitory effect on kinase activity [36]. However, these mutants have not been reported in lung cancer.

## ONCOGENIC EGFR SIGNALING KEY DOWNSTREAM PATHWAYS/ TARGETS

**Figure 2:**
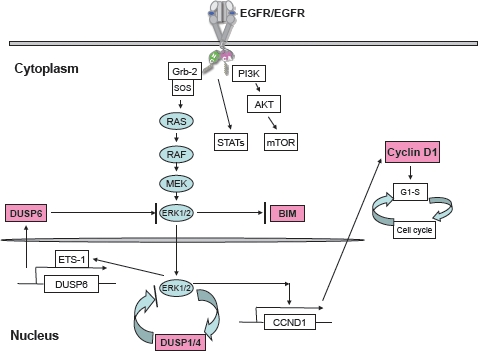
Key mediators downstream of EGFR signaling pathway in lung cancer EGFR dimerization results in autophosphorylation, kinase activation, and subsequent activation of three major signaling pathways,including RAS/RAF/MEK/ERK1/2, PI3K/AKT and STATs pathways. BIM is significantly induced to exhibit pro-apoptotic functions upon EGFR inhibition via mostly ERK regulation in NSCLC cells. Cyclin D1 is greatly suppressed by EGFR inhibition, promoting cell cycle arrest. ERK1/2 signalling is typically negatively regulated by a family of dual-specific MAPK phosphatases, known as DUSPs or MKPs, especially DUSP1, DUSP4, and DUSP6 in NSCLC. DUSP1 and DUSP4 function to terminate ERK signaling in nucleus whereas DUSP6 inhibits ERK activation in the cytoplasm.

Upon binding of natural ligands (e.g., EGF, TGFα, and multiple other ligands) to its extracellular domain, EGFR forms dimers with itself and other members of the ErbB family via specific dimerization domains [37,38], which induces conformational shifts that promote tyrosine autophosphorylation in the activation loop of EGFR and consequent kinase activation leading to stimulation of intracellular signaling cascades such as the RAS/RAF/ERK, PI3K/Akt, and STAT signaling pathways (Fig. 2). The EGFR family mediated signaling pathways have been shown to be important for proper regulation of many developmental, metabolic, and physiologic processes mediated by EGF, TGFα, and multiple other ligands. In numerous cancers, including glioblastomas, colon cancer, breast cancer, and non-small cell lung cancer, the output of the EGFR pathway is commonly increased by genetic mutation and overexpression of the receptor, overactivity of its ligands or cofactors and less commonly reduced inhibition through loss of its negative regulatory pathways driving the mitogenic, antiapoptotic, angiogenic and pro-invasive behaviour of the cancer cells. EGFR-targeted drugs including tyrosine kinase inhibitors, such as erlotinib and gefitinib, are primarily used in lung cancer treatment producing significant clinical responses in 10% to 30% of all NSCLC patients [32,39,40] and currently used as first line therapy for lung cancers with EGFR mutations achieving about 70% response rates [41,42]. Humanized monoclonal antibodies against the extracellular structure of EGFR such as cetuximab and panitumumab, are primarily used in colorectal cancer and head/neck cancer [43] and will not be further discussed in this review.

Inhibition of key signalling mediators downstream of EGFR should also lead to clinical effects in the treatment of lung cancer with robust EGFR activity. Therefore, identification and understanding critical downstream effectors of oncogenic EGFR variants should help to develop new treatment targets and in fact, a large number of pharmacological inhibitors against those key mediators are under intensive basic and clinical investigations as summarized below. The pivotal ERK1/2-MAPK and PI3K/AKT pathways play critical roles in gefitinib/erlotinib-induced antitumor effects in NSCLC cell lines and tumors with EGFR addiction [44,45]. However, inhibitors directly targeting ERK1/2 or PI3K/AKT have not been evaluated carefully in clinic yet.

### mTOR

mTOR is an important downstream effector of the PI3K/AKT signalling pathway and mTOR inhibitors can effectively block growth and survival signals by inactivating downstream effectors such as p70S6K and 4E-binding protein 1 [46]. mTOR represents an attractive target because its inhibition could allow avoidance of possible side effects associated with inhibition of upstream PI3K/AKT signaling molecules with broader biological functions, including those involved in glucose signaling [47].

### Bim

Bim, a proapoptotic BH3-only Bcl-2 family polypeptide and also known as BCL2-like 11, has been shown to be a key downstream effector of EGFR signalling by several groups [35,48,49]. Bim expression was significantly induced by EGFR TKI inhibition in gefitinib-sensitive EGFR-mutant lung cancer cells through both transcriptional and post-translational mechanisms. Knockdown of Bim by small interfering RNA was able to attenuate apoptosis induced by EGFR TKIs, and addition of a BH3 mimetic enhanced gefitinib-induced apoptosis, suggesting that inducing Bim or use of BH3 mimetics may give rise to similar effects to inhibition of EGFR by promoting apoptosis and even overcoming EGFR TKI treatment resistance in lung cancer.

### Cyclin D1

Cyclin D1 forms a complex with and functions as a regulatory subunit of CDK4 or CDK6, whose activity is required for cell cycle G1/S transition. Cyclin D1 has been identified as a key downstream effector of EGFR signalling by using microarray transcriptional profiling of gefitinib-resistant NSCLC EGFR L858R-T790M mutant H1975 cells exposed to the irreversible and in these cells still effective inhibitor, CL-387,785 versus gefitinib. Cyclin D1 was highly suppressed by CL-387,785 but not by gefitinib and downregulation of cyclin D1 resulted in suppression of E2F-responsive genes, consistent with proliferation arrest. EGFR-mutant NSCLC cells have higher expression of cyclin D1 than cells with wild-type EGFR and are sensitive to the cyclin-dependent kinase inhibitor flavopiridol [50]. Cyclin D1 has also been introduced as an important biomarker among EGFR, K-RAS and VEGFR in the BATTLE trial focusing on personalized therapy for lung cancer [51].

### Dual-specificity phosphatases

MAPK signalling is negatively regulated by a family of dual-specificity MAPK phosphatases, known as DUSPs or MKPs [52]. A nuclear-inducible DUSP1 has been reported to be a critical downstream effector of EGFR inhibition by AG1478 in PC9 cells. Downregulation of DUSP1 correlated with AG1478-induced apoptosis in PC9 cells via activation of JNK kinase activity, whereas overexpression of DUSP1 led to resistance to AG1478 of PC9 cells [53]. DUSP4 and DUSP6 have been well described as transcriptional targets of EGFR-ERK1/2 signalling and demonstrated as novel tumor growth suppressors in NSCLC [45,50,54]. In particular, genetically mediated loss of DUSP4 correlates closely with EGFR mutations suggestive of the cooperative nature of the two independent events. Due to their functional negative feedback roles in regulation of MAPKs, many DUSP family members may serve as potential targets for lung cancer therapy.

### Other targets

Some signalling pathways transduced by receptor tyrosine kinases other than EGFR may also play important roles in EGFR-addicted NSCLC and could serve as targets for therapeutic purpose. Recent studies have demonstrated close crosstalk between EGFR and MET [55]. Aberrant EGFR hyperactivation results in increased MET expression in EGFR-mutant NSCLC cells via HIF-1α activation but EGFR TKI resistance-rendering MET amplification could uncouple MET levels from the EGFR signalling pathway [56]. MET has been shown to be a key downstream mediator of EGFR-induced invasiveness in EGFR-dependent NSCLC cells, suggesting that therapeutic inhibition of MET in combination with EGFR blockade may prevent tumor metastasis beyond the effect of EGFR alone in a subset of lung cancers, in addition to the potential benefit of preventing the emergence of resistance through MET amplification [57].

## PRIMARY AND SECONDARY EGFR RESISTANCE

Primary and acquired drug resistances are key issues in the area of targeted therapeutics. Despite overexpression of EGFR in the majority of lung tumors, only a small fraction of patients responds significantly to EGFR inhibition and the majority of tumors demonstrate primary resistance. Activating mutations of EGFR correlate with exquisite sensitivity to growth inhibition by erlotinib or gefitinib, but patients ultimately develop progressive disease after a typical period of 6-12 months indicating the development of resistance to these agents [58].

## PRIMARY RESISTANCE

Primary resistance affects patients who are initially refractory to EGFR tyrosine kinase inhibitor treatment. Certain molecular factors have been identified as predictive of EGFR TKI response in lung cancers, such as increased EGFR gene copy number and activating mutations within the EGFR TK domain [30,59,60]. Thus, patients without these characteristics are more likely to present with primary resistance to EGFR TKIs. The recent IPASS study reported that Asian NSCLC patients containing wild-type EGFR had a shorter time to progression to EGFR TKIs as compared to the outcome of patients treated with classical chemotherapy and a very low response rate of 2% [41], suggesting that genetic wild-type of EGFR by and large confers primary resistance to EGFR TKIs.

### Resistant EGFR mutants

Multiple EGFR mutations have also been implicated in primary resistance to EGFR TKIs, such as the presence of insertion mutations in exon 20 of EGFR that precludes the binding of gefitinib or erlotinib to the EGFR TK domain conferring resistance [61]. Somatic exon 20 insertions are also detected in ErbB2 in NSCLC and similarly appear to confer resistance to gefitinib or erlotinib [61]. Even though the exon 20 insertions represent less than 5% of all known mutations in the EGFR gene, strategies aimed at overcoming resistance induced by exon 20 insertions of EGFR and ErbB2 have been studied by use of irreversible inhibitors of EGFR and ERBB2 as well as heat shock protein-90 inhibitors, in that interaction with HSP-90 seems to be required for stability of mutated EGFR and ErbB2 and HSP-90 inhibitors promote mutated EGFR degradation [62,63].

### K-RAS

K-RAS belongs to the RAS family of oncogenes and accounts for more than 90% of RAS mutations in NSCLC. K-RAS mutations have been detected in 15-30% of NSCLC, with the majority occurring in codons 12 and 13, in particular codon 12 (>90%). The mutations lead to impaired GTPase activity and subsequent constitutive activation of RAS signaling, which is downstream of EGFR leading to activation of proliferative and anti-apoptotic pathways such as the ERK signaling pathway. K-RAS mutations have been demonstrated to be significantly associated with primary resistance to EGFR-TKIs in a wide variety of tumor types including lung cancer [64-67]. K-RAS mutations present more commonly in adenocarcinomas from elderly patients and heavy smokers who have been identified as a group unlikely to respond to EGFR TKIs [68]. Development of effective K-RAS inhibitors remains one of the most daunting challenges for current tumor therapeutics.

### Other mechanisms

Other less clearly validated markers for primary resistance to EGFR TKIs include loss of PTEN, BRAF mutations, increased expression of MAPK, ABCG2, IGFR1, and BCL-2, and angiogenesis regulators [69]. Expression level of steroid receptor coactivator-3 (SRC-3) has recently been shown to inversely correlate with resistance to gefitinib or erlotinib in 48 NSCLC cell lines using the reverse-phase protein array technique, whereas high SRC-3 protein level correlates with resistance to the TKIs [70]. ALK translocations represented by EML4-ALK fusion are found to be mutually exclusive with EGFR or K-Ras mutations and predict for primary resistance to EGFR TKIs in patients with advanced NSCLC since EGFR output is not key to cell survival in these tumors [71]. An emerging concept about cancer stem cell (CSC) or cancer-initiating cells has been proposed as a potential mechanism of primary drug-resistance [72]. Signalling pathways involving TGF-β, Wnt, Notch, Hedgehog, PI3K/PTEN/mTOR, IGF-1R, histone demethylase, and histone deacetylase (HDAC) have been implicated in CSC self-renewal, maintenance, and plasticity [72,73]. It is postulated that any strategy aimed at killing the abundant non-stem cancer cells will fail without eradicating the few CSCs in a tumor. These cancer stem cells might be less dependent on growth pathways, such as the EGFR pathway and might survive drug inhibition. Acquired resistance is indeed hypothesized by some to emerge in this quiescent stem cell population over time by the acquisition of secondary mutations for example. Potentially those key regulators involved in the CSC programming may act as effective targets for drug development to overcome the primary resistance to anticancer drugs including resistance to EGFR-targeted therapy in lung cancer.

## ACQUIRED RESISTANCE

Acquired resistance generally affects patients who initially respond to treatment but subsequently experience a loss of response [74]. As EGFR TKIs are now proven as standard first-line therapy for NSCLC patients with EGFR mutations [41,42], a rapidly growing number of patients with acquired resistance will be encountered. Accordingly, a clinical definition of acquired resistance to EGFR TKIs has been established for unifying therapy and studying this subset of lung cancer [75].

### Secondary EGFR mutations

The acquisition of resistance to the targeted inhibition of kinases in cancer is by now a well-documented phenomenon in several cancer types. Although the importance of the cancer stem cell is firmly established for primary drug resistance, the etiology of acquired resistance is still the subject of some debate. As compared to the large number of secondary resistance mutations noted in acquired imatinib resistance in CML, in the case of EGFR-TK, there are currently only several documented resistance point mutations to gefitinib and erlotinib, including T790M [32,33], L747S [35] and D761Y [34]. The T790M point mutation in the EGFR kinase domain has been reported to be the most common secondary resistance mutation, accounting for about 50% of tumors relapsed from prior TKI therapy [33]. The T790M mutation results in alteration of the topology of the ATP-binding pocket not only interrupting the physicochemical binding of gefitinib/erlotinib, but also leading to much increased affinity of the EGFR protein to ATP resulting in resistance to EGFR-TKIs [76]. Resistance to the T790M mutation in lung cancer could be overcome in vitro by irreversible EGFR small molecule inhibitors such as CL-387,785 [77] and BIBW2992 [78], Hsp90 inhibition [79], combination of multiple RTK inhibitor and mTOR inhibitor [80], combination of TS-targeting drugs (5-fluorouracil or pemetrexed) and BIBW2992 [81], and novel mutant-selective EGFR kinase inhibitors [82].

### MET amplification

The second major mechanism of acquired resistance reported is the amplification of the MET oncogene that activates ERBB3/PI3K/AKT signalling in lung cancer [57]. MET amplification was found in 4 of 18 lung cancer biopsy samples obtained from patients with acquired resistance to gefitinib or erlotinib [57]. Preclinical data suggests that combination of EGFR and MET TKIs can be a treatment strategy for EGFR mutated NSCLC either delaying acquired resistance or for the treatment of tumors with co-existing EGFR activating mutations and MET amplification [83,84].

### Other mechanisms

Given that T790M and MET amplification collectively account for approximately 60% of the acquired resistance cases, there are clearly additional mechanisms that underlie resistance to EGFR TKIs. Other mechanisms that have been implicated in acquired resistance include overexpression of AXL tyrosine kinase receptor [85], altered EGFR trafficking [86], expression of insulin-like growth factor-1 [87], amplification of mutant EGFR or hyperactivation of components of downstream signaling pathways [88], and expression of the ABCG2 drug-efflux transporter [89]. Recently, it has been shown that activation of TGF-β/IL-6 signaling leads to epithelial-to-mesenchymal transition and erlotinib resistance [90]. Targeting key EGFR-downstream signalling pathways should be an alternative approach for overcoming resistance to erlotinib or gefitinib in lung cancers. For example, the mTOR inhibitor, everolimus, has been shown to reduce the expression of EGFR signalling effectors and cooperates with gefitinib to overcome resistance [91], and the combination of an mTOR inhibitor and an irreversible EGFR inhibitor may be an effective strategy to overcome EGFR TKI resistance.

## STRUCTURAL IMPLICATIONS OF EGFR ACTIVATION/ STRUCTURAL CONSEQUENCES OF ONCOGENIC EGFR MUTATIONS

### EGFR activation

Normal regulation of EGFR family receptor tyrosine kinases comprises a precise orchestration of interconnecting components. Acquired mutations (Fig. 1), even of single amino acids, can deleteriously alter the choreography of regulation; however, it is these acquired mutations that provide a therapeutic entry-point for targeted inhibition of dysregulated EGFR signaling. Regulation of EGFR family signal transduction is one of the most comprehensively studied of the receptor tyrosine kinase family at the atomic-level, and current structural studies are still providing surprising and exciting new information about their regulation. Principal among these recent findings is the discovery that an asymmetric homodimer assembly is critical for kinase activation. This, alongside studies investigating the structures of activating and resistance mutations in the kinase domain itself, has recently provided a far clearer understanding of the mechanisms of EGFR family activation and resistance to small molecule inhibitors.

The catalytic portion of the EGFR family comprises a cytoplasmic domain with a protein kinase fold. This fold generally functions to catalyze phosphotransfer of the ATP γ-phosphate to target protein substrates primarily on tyrosine, serine and threonine residues. The protein kinase fold is a bi-lobed domain that includes a C-terminal lobe rich in alpha-helices and an N-terminal lobe that consists mainly of beta-strands. In the transition between inactive and active states for protein kinases, conformational changes usually occur in the N-terminal lobe that reposition the catalytic residues to the correct spatial locations that favor phosphotransfer. Conformational changes associated with activation also often occur within a short region of the kinase domain termed the activation segment. Autoinhibitory conformations of protein kinases are well described by structural biology studies and have proven to be diverse among the family; however, the spatial orientation of residues required for catalytic competency is very well conserved. While the structural diversity of inactive states among protein kinases provides well-documented therapeutic targets (imatinib targets an inactive conformation of BCR-Abl), structural similarities in kinase active state conformations can lead to difficulties in achieving specificity in kinase-targeted drug discovery. In a general sense, the acquisition of transforming point mutations for this class of proteins disrupts the normal active-inactive balance to favor the active state.

**Figure 3:**
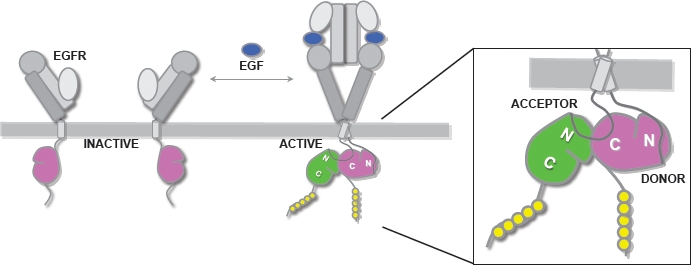
Schematic diagram of EGFR activation Shown for EGFR are the four domains in the extracellular region, transmembrane helix, and the cytoplasmic juxtamembrane region and tyrosine kinase domain. In the absence of ligand, the EGFR resides on the cell surface in an inactive/autoinhibited conformation (left). Upon ligand (EGF) binding, the autoinhibitive conformation in the EGFR ectodomain is released, leading to ectodomain-mediated receptor dimerization (right). In the cytoplasm, receptor dimerization results in formation of an asymmetric kinase homodimer in which the C-terminal lobe of the “donor” kinase (colored pink) interacts with the N-terminal lobe of the acceptor/activated kinase (colored green) to confer allosteric activation of the acceptor kinase. The juxtamembrane segment of the acceptor kinase in turn associates with the C-terminal lobe of the donor kinase to stabilize this activating asymmetric dimer (right zoom).

In the EGFR family of receptor tyrosine kinases, the specific mechanisms of normal kinase regulation are now well-defined by structural biology techniques [92,93]. Extracellular conformational rearrangement upon ligand (e.g. EGF) binding allows dimerization of the receptor, the consequence of which is the ability of the cytoplasmic kinase domains to trans-activate (Fig. 3). Activated EGFR kinase is able to autophosphorylate the C-terminal tail, thereby creating recruitment sites for phosphotyrosine-binding domains of downstream proteins in EGFR signaling cascades. Until recently the atomic-level mechanisms of EGFR trans-activation were not known; however, analysis of the crystal packing within previous EGFR kinase domain crystal structures led to the observation that in the active state the N-terminal lobe of the EGFR kinase domain interacts with the C-terminal lobe of a partner kinase domain [94]. Subsequent studies have validated this tail-head, or donor-acceptor, interaction and discovered that the donor, or tail, molecule activates the acceptor, or head, molecule by inducing an activating conformational movement centered on the N-terminal lobe [95,96]. The result is that only one of the kinase domains in an activated EGFR-family receptor complex is in a catalytically competent state, a finding that helps explain the mechanism of ErbB2 activation by the catalytically inactive ErbB3 via heterodimerization. Further studies have shown that the juxtamembrane region (the segment connecting the transmembrane helix to the kinase domain) of the active/acceptor EGFR stabilizes this asymmetric dimer by interacting with the C-terminal lobe of the donor kinase [16,97]. Acquired point mutations in the juxtamembrane region seen in NSCLC (e.g. V689M, L703F) further promote the asymmetric head-tail active state [16], providing a clear rationale for activated EGFR in these patients.

### Oncogenic EGFR mutations

Structural studies have also provided clues to the mechanisms by which activating and resistance mutations alter kinase activity and how small molecule inhibitors can specifically target mutant enzyme [98]. The most commonly seen activating point mutation in EGFR, L858R, is incompatible with the inactive state of the kinase [24,99], and the crystal structure of the L858R mutant revealed that additional hydrogen bonds are formed which serve to further stabilize the active state of the kinase [99]. The mechanism by which this and other mutations activate EGFR, however, is not completely explained by a conformational predisposition to the active-state. Kinetic analyses found that clinically relevant mutations in EGFR alter binding to both ATP and inhibitors (e.g. erlotinib and imatinib) in such a way that the ratio of *K*_d_ to *K*_m[ATP]_ is altered in favor of the inhibitors [98-100]. These measured changes in apparent *K*_i_ therefore provide a mechanism for selective inhibition of mutant EGFR by small molecules such as erlotinib and imatinib [98]. Alterations in ATP-dependent reaction rates and inhibitor binding affinities are probably the mechanism for acquired resistance by the T790M mutation [76]. Therapeutically, the use of covalently binding inhibitors (e.g. HKI-272, BIBW2992, PF00199804) may present a mechanism to overcome resistance by binding in a similar fashion to non-covalent inhibitors, but with covalent attachment to EGFR residue cysteine-797 [82,98].

## THE CLINICAL USE OF EGFR-TARGETED AGENTS IN NON-SMALL CELL LUNG CANCER

### Initial studies

Over the last few years, we have seen a revolution in the understanding of the appropriate use of EGFR-targeted therapy in non-small cell lung cancer. Initial studies of both erlotinib and gefitinib demonstrated good overall tolerability with skin rashes, diarrhea and occasional episodes of pneumonitis noted as the main concerns and modest activity of 10-20% response rates were noted in unselected populations [101]. Significantly higher responsiveness was noted in certain patient subsets, such as patients with adenocarcinoma histology, women, patients of Oriental descent and non-smoking patients. Disappointingly, four large randomized studies combining these drugs with upfront chemotherapy demonstrated negative results while in the second/third-line setting an overall survival benefit was noted in the pivotal BR.21 study of erlotinib versus best supportive care [102] but not in the ISEL study comparing gefitinib with best supportive care [103], although an overall survival benefit in the Asian subset was observed. These studies ultimately led to the approval of erlotinib in the U.S. and gefitinib in many Asian countries for second-line or subsequent use.

### Search for predictive biomarkers

Alongside these key clinical studies, multiple biomarkers were identified and tested, most notably EGFR expression by immunohistochemistry, EGFR copy number changes detected by FISH or quantitative PCR and with the discovery of activating EGFR mutations in highly responsive patients, EGFR mutational status [104]. Lately, it has become quite clear that the best predictor of a major clinical response is the presence of activating EGFR mutations in the tumor, mainly exon 19 deletions or L858R mutation. Indeed, at this point it needs to be recognized that EGFR-mutant lung adenocarcinoma is a distinct clinical entity and currently upfront general testing for EGFR mutational status is endorsed by many leading institutions, is available through several commercial entities and with the use of multiple platforms ranging from direct sequencing to high sensitivity, mutation-specific detection techniques [105]. EGFR copy number changes also have some predictive value but most of its value might lie in the fact that true EGFR gene amplification typically closely correlates with EGFR mutational status and thereby is a surrogate marker for such. EGFR gene copy number increase (polysomy) without EGFR gene amplification is much less robust of a predictor. EGFR expression by immunohistochemistry has not proven to be a clinically effective predictor of responsiveness. Overall, lately there has been a dramatic shift in clinical practice towards the isolated use of EGFR mutational status when choosing EGFR-targeted therapy based on numerous first-line clinical studies listed below. Among other biomarkers, serum proteomics have also been developed and in a number of studies have shown correlation with clinical benefit from EGFR TKI therapy. An approved test (VeriStrat) is available for clinical use but given other available markers, its clinical utility remains somewhat ill-defined [106]. K-RAS mutational status repeatedly have been shown to be a negative predictor of responsiveness and can be used as a “negative surrogate” for EGFR mutational status since by and large these mutations are exclusive of each other [65].

### First-line use

Based on the poor overall outcome and significant toxicity of upfront chemotherapy in advanced non-small cell lung cancer, EGFR TKI therapy as front-line treatment has significant appeal for the appropriate patient. Initial studies focusing on leveraged patient populations based on clinical predictors of higher EGFR TKI responsiveness or selection by EGFR mutational status suggested potentially excellent activity with response rates in the 50-90% range in patients with tumors harboring activating EGFR mutations. The American iTARGET trial focused on a clinically enriched population of chemo-naïve patients with non-squamous histology and demonstrated a 55% RR, PFS of 9.2 months and OS of 17.5 months in EGFR mutation positive patients [107]. The Spanish study group reported the results of a prospective phase II study about the use of erlotinib in advanced NSCLC patients harbouring EGFR mutations. 2,105 patients were screened and 350 (16.6%) identified to carry EGFR mutations [42]. Median PFS and OS for the 217 patients who received erlotinib were 14 and 27 months, patients with L858R had longer PFS than patients with exon 19 deletions and outcomes did not seem to differ whether erlotinib was given in the first or second-line setting. A combined survival analysis (I-CAMP) of seven prospective Japanese trials of 148 patients with EGFR mutations who received gefitinib demonstrated a response rate of 76.4%, median PFS of 9.7 months and overall survival of 24.3 months [108]. Good performance status and chemotherapy-naïve status were significantly associated with a longer progression-free survival. On the other hand, overall survival was not affected by first-line or second-line gefitinib use suggestive of the benefit to be sustained through several lines of therapy.

Recent, randomized clinical studies have brought further clarity to this field. The IPASS study enrolled 1,217 chemotherapy-naïve patients with advanced lung adenocarcinoma with no or light smoking history and a PS of 0-2 [41]. Patients were randomized to receive gefinitib versus carboplatium/paclitaxel for a maximum of 6 cycles. Gefitinib demonstrated superiority in terms of PFS for the ITT population with a HR of 0.74 (12-months PFS of 24.9 versus 6.7%), however the hazard ratio was not constant over time. Further review showed dramatic separation of outcomes based on EGFR mutant status. In the EGFR-mutant group (59.7% of all patients with available test result) the objective response rate of 71.2% and the PFS of 9.5 months in the gefitinib group was much superior to an ORR of 47.3% and PFS of 6.3 months with chemotherapy compared to a sobering 1.1% ORR and a 1.5 month PFS with gefitinib which was much worse than results with standard chemotherapy (hazard ratio of 2.85) in wild-type patients. The results of the First-SIGNAL study comparing first-line cisplatinum/gemcitabine with gefitinib in the first-line treatment of 309 Asian never-smokers with advanced adenocarcinoma similarly showed improved 1-year PFS with gefitinib and a response rate of 84.6 versus 37.5% in EGFR mutation positive patients while extremely poor results were noted with gefitinib in wild-type patients [109]. These non-mutant selective studies demonstrated that clinical factors are less predictive of responsiveness than tumor genetics and provide very strong justification for upfront testing if first-line EGFR therapy is contemplated since clinically selected but EGFR WT patients appear to fare dramatically worse on gefitinib than chemotherapy.

Recently, the results of studies exclusively focusing on EGFR-mutated adenocarcinoma have also been reported. The WJTOG3405 study enrolled 177 chemotherapy-naïve patients aged 75 years or younger and diagnosed with stage IIIB/IV non-small cell lung cancer or postoperative recurrence harboring EGFR mutations- either exon 19 deletions or L858R [110]. Patients were randomly assigned to gefitinib or cisplatinum/docetaxel for 3-6 cycles. The gefitinib group had significantly longer progression-free survival compared to chemotherapy (9.2 versus 6.3 months). Myelosuppression, alopecia, fatigue were more common with chemotherapy, while skin toxicity, liver dysfunction and diarrhea were more frequent in the gefitinib group. Two patients in the gefitinib group developed interstitial lung disease (2.3%). The NEJ002 study [111] was prematurely closed after accruing 230 patients due to a significant benefit seen for gefitinib versus carboplatinum/paclitaxel in patients with prospectively identified EGFR-mutated advanced non-small cell lung cancer. Analysis of the first 200 patients showed a doubling of PFS by gefitinib (10.8 vs 5.4 months). Overall response rates were 74% with gefitinib and 31% with chemotherapy. Median survival was 30.5 months vs 23.6 months with gefitinib versus chemotherapy, the OS difference was not statistically significant. These results are almost superimposable with each other and further demonstrate the excellent activity of EGFR TKIs in this setting. Inoue and colleagues [112] completed a phase II trial of gefitinib in patients with poor PS harboring EGFR mutations and a RR of 66%, PFS of 6.5 and OS of 17.8 months were seen demonstrating very impressive outcomes in a patient population with a generally very poor survival redefining the boundaries of when treatment might still be beneficial since patients with a PS of >2 are generally not considered to be candidates for chemotherapy.

### Maintenance therapy

In the SATURN trial, 889 patients with advanced non-small cell lung cancer and no evidence of disease progression after 4 cycles of chemotherapy were randomized to receive erlotinib versus placebo until progression or unacceptable toxicity [113]. PFS (the primary endpoint) was prolonged in the erlotinib group (HR 0.71) and all biomarker groups showed a PFS benefit with erlotinib. In particular, EGFR mutant patients saw a marked improvement in PFS with erlotinib therapy (HR 0.10). Median OS was also significantly improved for the total population in the erlotinib group (HR 0.81). The survival benefit was particularly large in patients with adenocarcinoma histology and was not driven by the EGFR-mutation positive subgroup, with a significant improvement in survival observed in the EGFR wild-type group ultimately leading to FDA-approval of erlotinib in this indication. Notably, pemetrexed is also approved as maintenance therapy in advanced non-squamous non-small cell lung cancer and bevacizumab is also utilized in the same setting until disease progression in bevacizumab-eligible patients based on the survival benefit seen in the ECOG4599 study confounding this field. Erlotinib certainly appears to be an excellent choice in the maintenance setting in patients with EGFR-mutated tumors who have not received it as first-line therapy.

### Second-line therapy

The pivotal BR.21 study which led to the approval of erlotinib randomized 731 chemotherapy-refractory patients with advanced non-small cell lung cancer to erlotinib or placebo in a 2:1 ratio and a response rate of 8.9% was seen in the erlotinib group and an overall survival benefit of 6.7 versus 4.7 months was noted [102]. ISEL was a randomized, placebo-controlled, international multicenter phase III trial comparing gefitinib versus BSC as second or third-line treatment for patients with advanced NSCLC. 1,692 patients were enrolled in a ratio of 2:1 to receive gefitinib 250 mg daily or placebo plus BSC [103]. Differences in median survival did not reach statistical significance while a higher response rate and TTP was noted in the gefinitib arm. On preplanned subgroup analyses, a longer survival time was seen for never-smoker and Asian patients (9.5 vs 5.5 months) treated with gefitinib. Patients with EGFR mutations had a higher response rate than wild-type patients (37.5 vs 2.6%). The INTEREST trial compared gefitinib with docetaxel as second or third-line therapy in 1,466 patients with advanced NSCLC treated with prior platinum-based chemotherapy [114]. Median OS was 7.6 months in the gefitinib and 8.0 months in the docetaxel arm demonstrating non-inferiority of gefitinib as compared to docetaxel. Of note, EGFR mutation positive patients had longer PFS and higher RR (42.1 vs 21.1%) and patients with high EGFR copy number also had higher RR (13% vs 7%) with gefitinib as compared with docetaxel. The Korean ISTANA trial compared gefitinib with docetaxel as second-line treatment in 161 patients with advanced NSCLC and PFS HR (0.729), 6-months PFS (32 vs 13%) and RR were found to be improved with gefitinib when compared with docetaxel while OS was not different [115].

### Maintenance beyond progression

Riely and colleagues [116] reported that a subset of patients with non-small cell lung cancer who had acquired resistance to EGFR TKIs and had discontinued treatment progressed rapidly as shown by increased SUV in PET scans at 3 weeks follow-up consistent with a disease-flare associated with reduction of treatment pressure of a known biological pathway. This has led to the unproven practice of continuing EGFR TKI in primarily EGFR TKI-sensitive patients at the time of disease progression. This issue has significant implications for clinical practice and at least one study is ongoing to answer the question of whether this practice is beneficial or not (Table 1).

**Table 1:** Representative ongoing clinical studies focusing on EGFR-mutant lung tumors/EGFR inhibition. Higher Quality Version

Identifier	Biomarker	Study type	Disease setting	Treatment	Endpoint	Sponsor
Nct0949650	EGFR activating mutation	Phase III, prospective, randomized	Stage IIIB or IV Adenocarcinoma of the Lung w/ an EGFR Mutation	BIBW 2992 vs. Chemotherapy as First-line	Progression-free survival	Boehringer Ingelheim
Nct00567359	EGFR mutation	Phase II	Resected Stage IA-B, IIA-B, or IIIA NSCLC with EGFR mutation	Adjuvant Erlotinib	2 year disease free survival	MGH
Nct00577707	EGFR mutation	Prospective, phase II	Stage IA-B, IIA-B, or IIIA NSCLC with EGFR mutation	Preoperative Cisplatinum/pemetrexed/ erlotinib, then erlotinib for 2 years postop	Pathological CR	MSKCC
Nct00660816	EGFR TKI responsive with secondary progression	Randomized phase II	Stage IIIB/IV NSCLC	Pemetrexed versus pemetrexed + erlotinib	Progression-free survival	Case Western Reserve University
Nct01085136	Unselected	Phase II, randomized	Stage IIIb/IV NSCLC	BIBW 2992 followed by comparator chemotherapy alone or paclitaxel + BIBW2992 on progression	Overall survival	Boehringer-Ingelheim
Nct00503971	EGFR mutant	Phase I/II	Stage IIIB/IV NSCLC	Erlotinib+ vorinostat	Phase I: maximum tolerated dose Phase II: safety and efficacy (RR)	Spanish Lung Cancer Group
Nct01167244	EGFR mutant or EGFR TKI responsive	Phase II	Stage IIIB/IV NSCLC	BMS-690514	Overall RR	Bristol-Myers-Squibb
Nct01068587	No response to prior chemo	Phase I/ randomized phase II	Stage IIIb/IV NSCLC	Erlotinib vs erlotinib+ GSK1363089	Recommended phase II dose Safety, efficacy (RR, clinical benefit)	NCIC/GlaxoSmithKline
Nct00596648	Clinical benefit on erlotinib	Phase I/ randomized phase II	Stage IIIb/IV NSCLC	XL184 vs XL184+ erlotinib	Recommended phase II dose Safety, efficacy	Exelixis
Nct00769067	Unselected	Randomized phase II	Stage IIIb/IV NSCLC	PF-00299804 vs erlotinib	Efficacy tolerance	Pfizer
Nct00854308	Unselected	Randomized phase II	Stage IIIb/IV NSCLC	Erlotinib+ MetMab vs erlotinib	Activity Safety	Genentech
Nct00826449	Unselected	Phase I/II	Stage IIIb/IV NSCLC	Erlotinib+ dasatinib	Recommended phase II dose Anti-tumor activity (PFS)	MDACC
Nct00548093	Unselected, 3^rd^ line after erlotinib failure	Phase II	Stage IIIb/IV NSCLC	PF-00299804	Anti-tumor activity (ORR)	Pfizer
Nct00965731	Unselected, 2^nd^ line	Phase I/II	Stage IIIb/IV adenocarcinoma	Erlotinib alone vs erlotinib + PF-02341066	Safety Anti-tumor activity	Pfizer

### Locally advanced non-small cell lung cancer

With proven benefits of EGFR TKIs in the metastatic setting, it would seem logical that such benefits would extend to earlier stages of the illness. Nonetheless, the SWOG0023 study surprisingly demonstrated inferior outcomes with maintenance gefitinib versus placebo following definitive chemoradiotherapy in patients with locally advanced non-small cell lung cancer [117]. Notably, these patients were not selected by biomarker status. Few current studies focus on exploring EGFR TKI therapy in this setting.

### Adjuvant therapy

EGFR TKIs provide mostly palliative benefit in the advanced setting similar to the benefit of Herceptin in metastatic breast cancer. In patients with resected lung cancer, the hope is that this class of drugs would on the other hand improve cure rates and studies in this setting are eagerly awaited. The RADIANT study is a phase III study comparing erlotinib with placebo in resected stage IB-IIIA NSCLC patients with EGFR IHC or FISH-positive tumors with the primary endpoint of improvement in DFS. Efficacy data are eagerly awaited, it has been reported that 12% of all samples carry EGFR mutations and 19% K-Ras mutations [118]. A single-arm adjuvant study focusing purely on EGFR-mutated tumors thereby examining a more enriched population is also ongoing (Table 1).

## ACQUIRED RESISTANCE

### Irreversible EGFR inhibitors

The most common acquired resistance mechanism is the emergence of EGFR-T790M, notable in about 50% of EGFR TKI-responsive patients at the time of disease progression. Prevention or overcoming resistance mediated by EGFR T790M is one of the most important and challenging research tasks in this field [58]. While in vitro multiple irreversible EGFR inhibitors have been noted to retain at least partial efficacy against EGFR T790M, initial experience with the irreversible dual EGFR/ErbB2 TKI, neratinib (HKI-272) was disappointing [119]. Another promising irreversible dual EGFR/ErbB2 inhibitor, BIBW2992 continues to generate interest. Results of the phase II LUX-Lung-2 study focusing on patients with EGFR-mutated non-small cell lung cancer have been reported and demonstrated a 61% response rate, PFS of 14 months and median survival of 2 years [120]. Phase III studies of this compound in multiple settings, including following failure of erlotinib or gefitinib are ongoing. PF00299804, an irreversible HER1, 2 and 4 inhibitor has also shown preliminary anti-tumor activity [121] and a predictable safety profile in a phase II study in patients with NSCLC after failure of chemotherapy and erlotinib. Several responses as well as prolonged stable disease were reported in erlotinib-refractory patients suggestive of potential clinical activity in this subset[122]. Further studies of this compound are also ongoing. One major concern about these compounds is whether the therapeutic window might be too narrow in this setting and side effects as a result of WT EGFR or ErbB2 inhibition might be limiting. Recently, through a targeted chemical screen selective inhibitors against T790M have been reported [82] and there is certainly hope that such rationally designed compounds might ultimately provide sufficient selectivity.

### MET inhibition

At least in some, possibly as often as in 20% of tumors, acquired resistance might be mediated by overamplification of the MET oncogene rewiring oncogenic pathways through overtaking activation of the key coupler, ErbB3. Data also suggests that in some tumors, MET-amplified tumor cells might preexist and ultimately emerge as the predominant clone [83]. These data might suggest that combination strategies of EGFR and MET inhibition either at the outset to prevent or at the time of progression to overcome resistance could be promising and multiple clinical studies with a wide range of MET-targeted agents are ongoing. At least one study has demonstrated prolonged PFS with the combination of erlotinib with the MET TKI, ARQ197 as compared to erlotinib alone [123] and phase III studies in the EGFR TKI-naïve setting are ongoing.

### Other strategies

Several preclinical reports showed that other agents, such as the anti-EGFR monoclonal Ab cetuximab or PI3K/mTOR inhibitors combined with irreversible EGFR inhibitors hold promise to overcome resistance mediated by T790M [124]. Heat shock protein inhibitors such a geldanamycin or 17-DMAG are also thought to be a potent strategy against T790M [79].

### Novel biomarkers

Both primary and acquired resistance turn out to be quite complex biologically and generate a tremendous need for appropriate biomarkers both for treatment selection as well as monitoring. Novel platforms for the detection of circulating tumor cells and genetic changes in these tumor cells seem the most promising to fill this void. E.g. one study of CTCs from lung cancer patients was able to identify EGFR T790M in CTCs of some patients and progression-free survival was shorter as one might expect in patients with than without T790M on erlotinib [125].

## ABBREVIATIONS USED:

EGFR: epidermal growth factor receptor
TKI: tyrosine kinase inhibitor
OS: overall survival
RR: response rate
BSC: best supportive care
NSCLC: non-small cell lung cancer
PFS: progression-free survival
HR: hazard ratio
ORR: overall response rate
CR: complete remission
